# Supplementary figures and images for: Morphofunctional changes in the immune system in colitis-associated colorectal cancer in tolerant and susceptible to hypoxia mice
Source: PeerJ. 2025 Feb 25;13:e19024. doi: 10.7717/peerj.19024 (PMC11869898; doi:10.7717/peerj.19024)

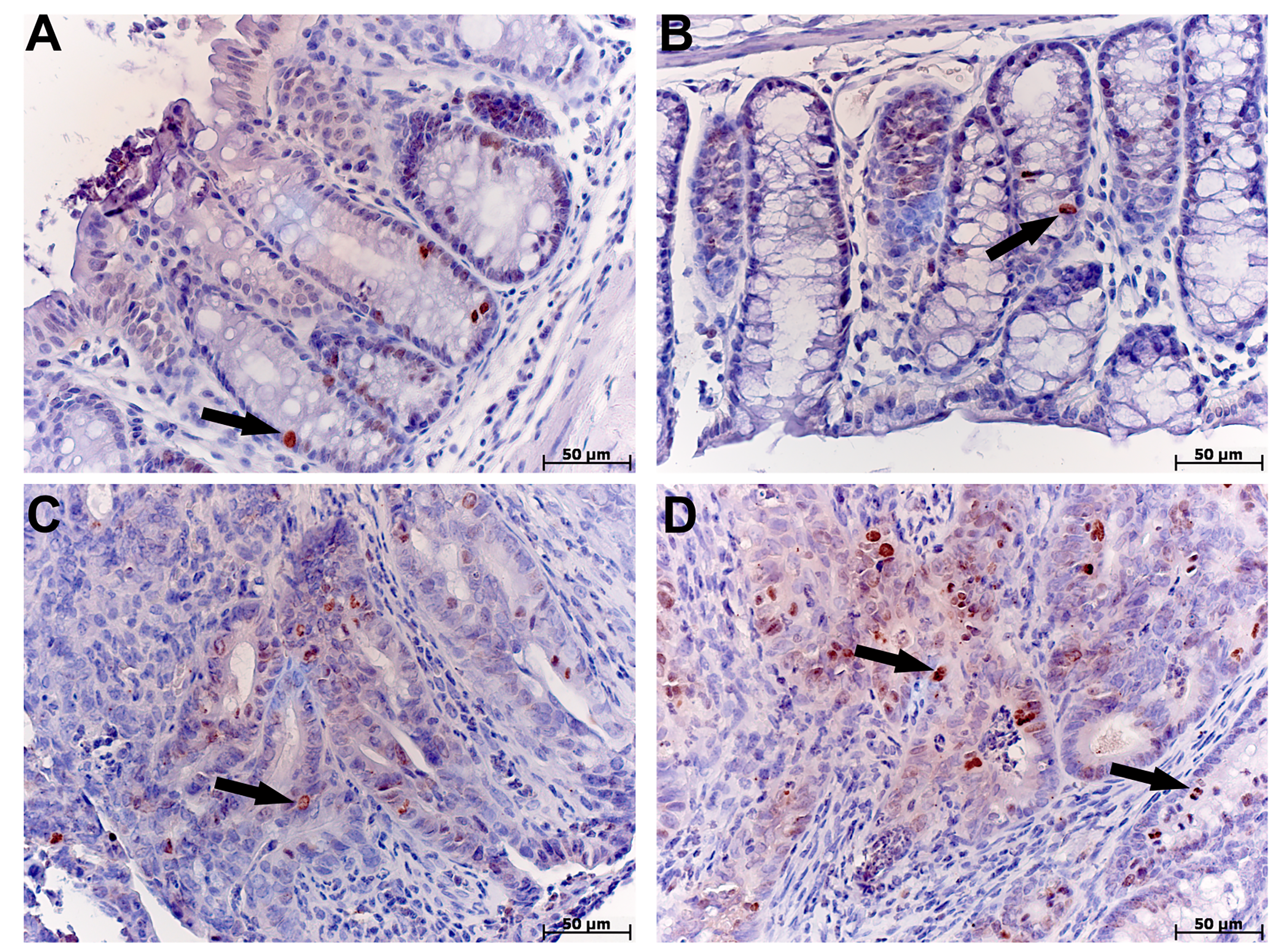

Supplement: Supplemental Information 1 — Staining with antibodies to Ki-67. (A) tolerant to hypoxia mice, control group, single Ki-67 positive cells (arrow); (B) susceptible to hypoxia mice, control group, single Ki-67 positive cells (arrow); (C) tolerant to hypoxia mice, experimental group, glandular intraepithelial neoplasia, Ki67 positive cells are detected among tumor cells (arrow); (D) susceptible to hypoxia mice, experimental group, adenocarcinoma, among the tumor cells there are many Ki-67-positive ones (arrows) [file peerj-13-19024-s001.png]
