# Supplementary material for: Morphofunctional changes in the immune system in colitis-associated colorectal cancer in tolerant and susceptible to hypoxia mice
Source: PeerJ. 2025 Feb 25;13:e19024. doi: 10.7717/peerj.19024 (PMC11869898; doi:10.7717/peerj.19024)
Supplement: Supplemental Information 4 [file peerj-13-19024-s004.pdf]

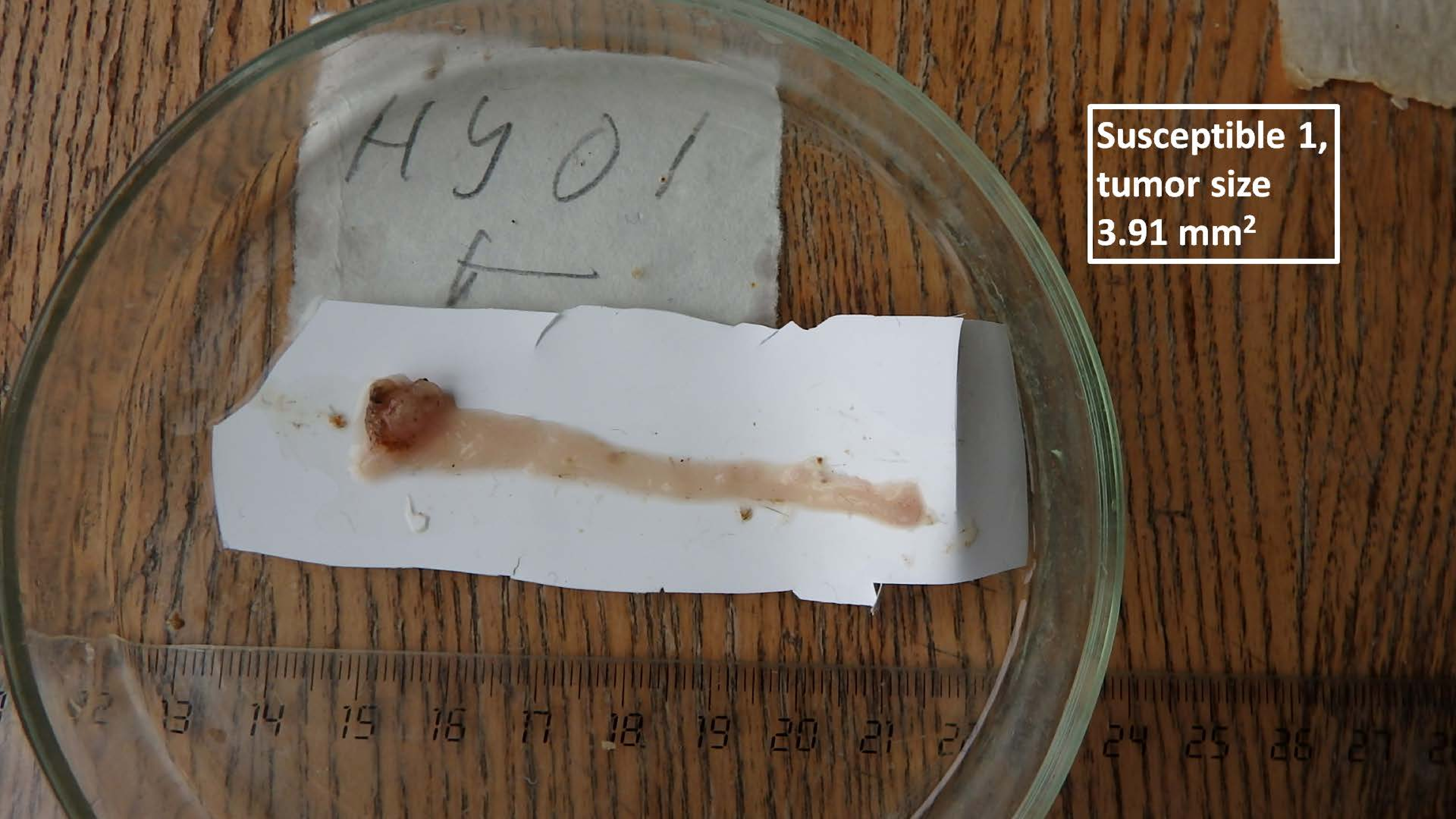
HY01
Susceptible 1, tumor size 3.91 mm²

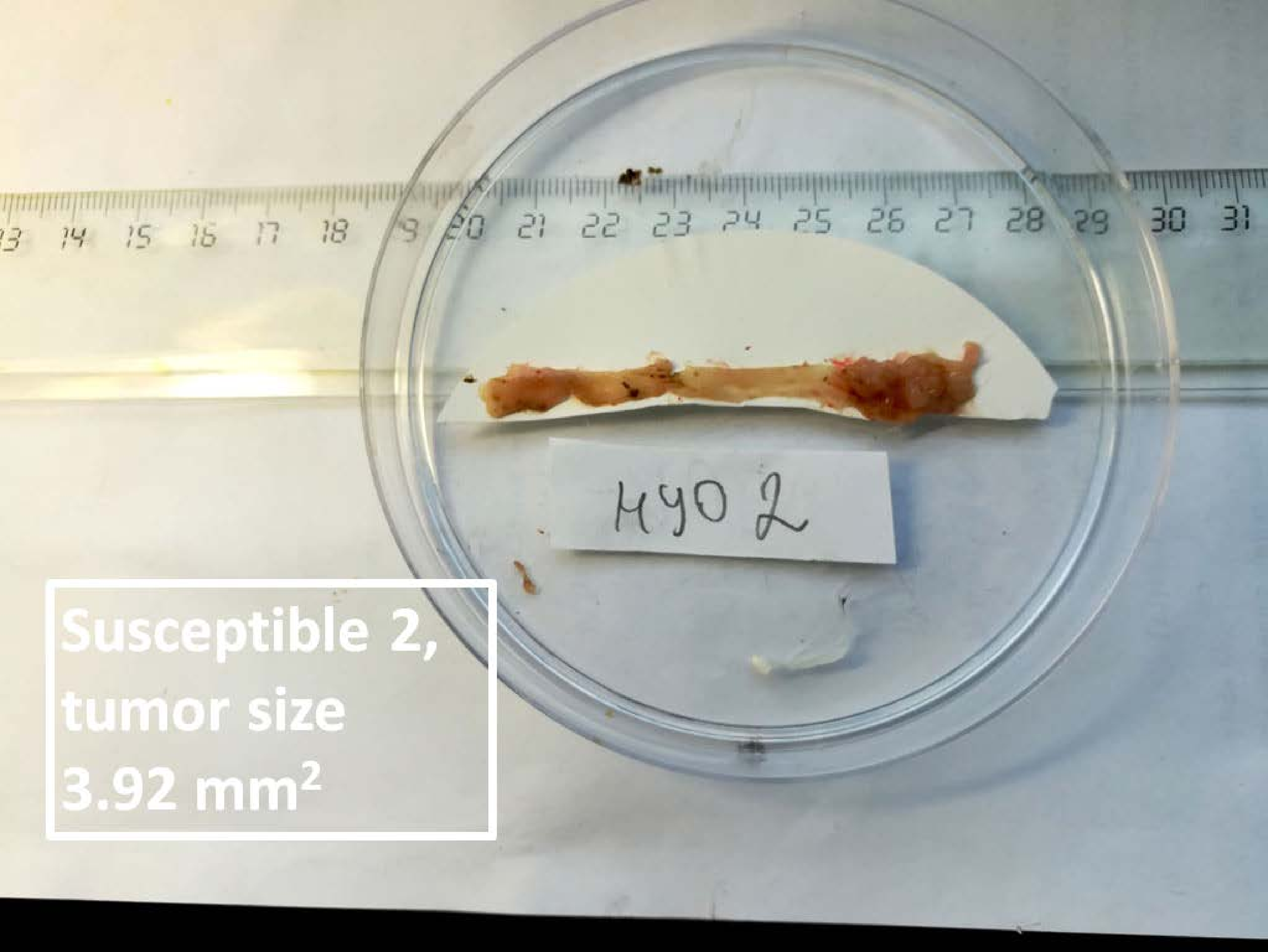
Susceptible 2,
tumor size
3.92 mm²
HYO 2

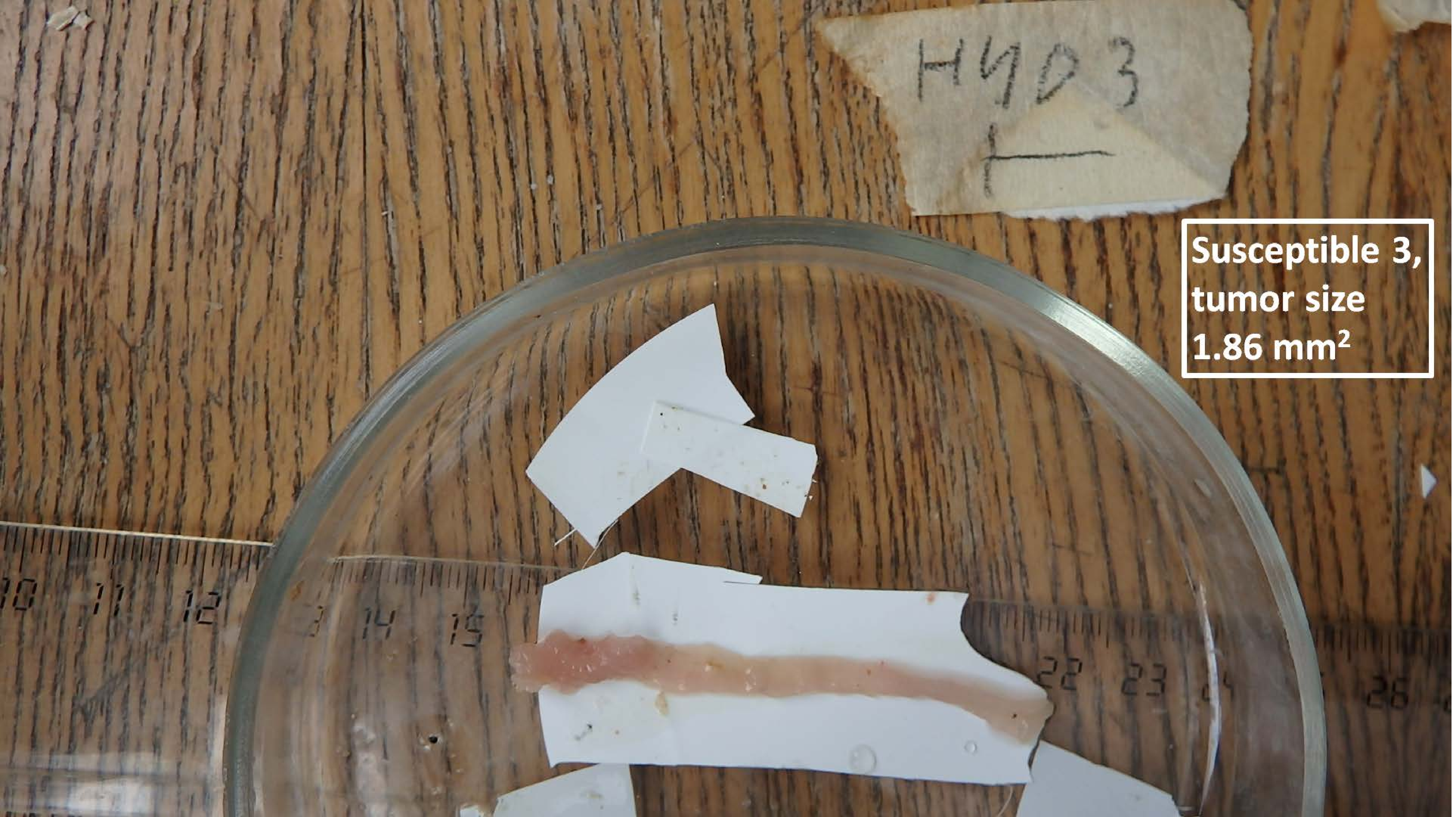
Susceptible 3,
tumor size
1.86 mm²

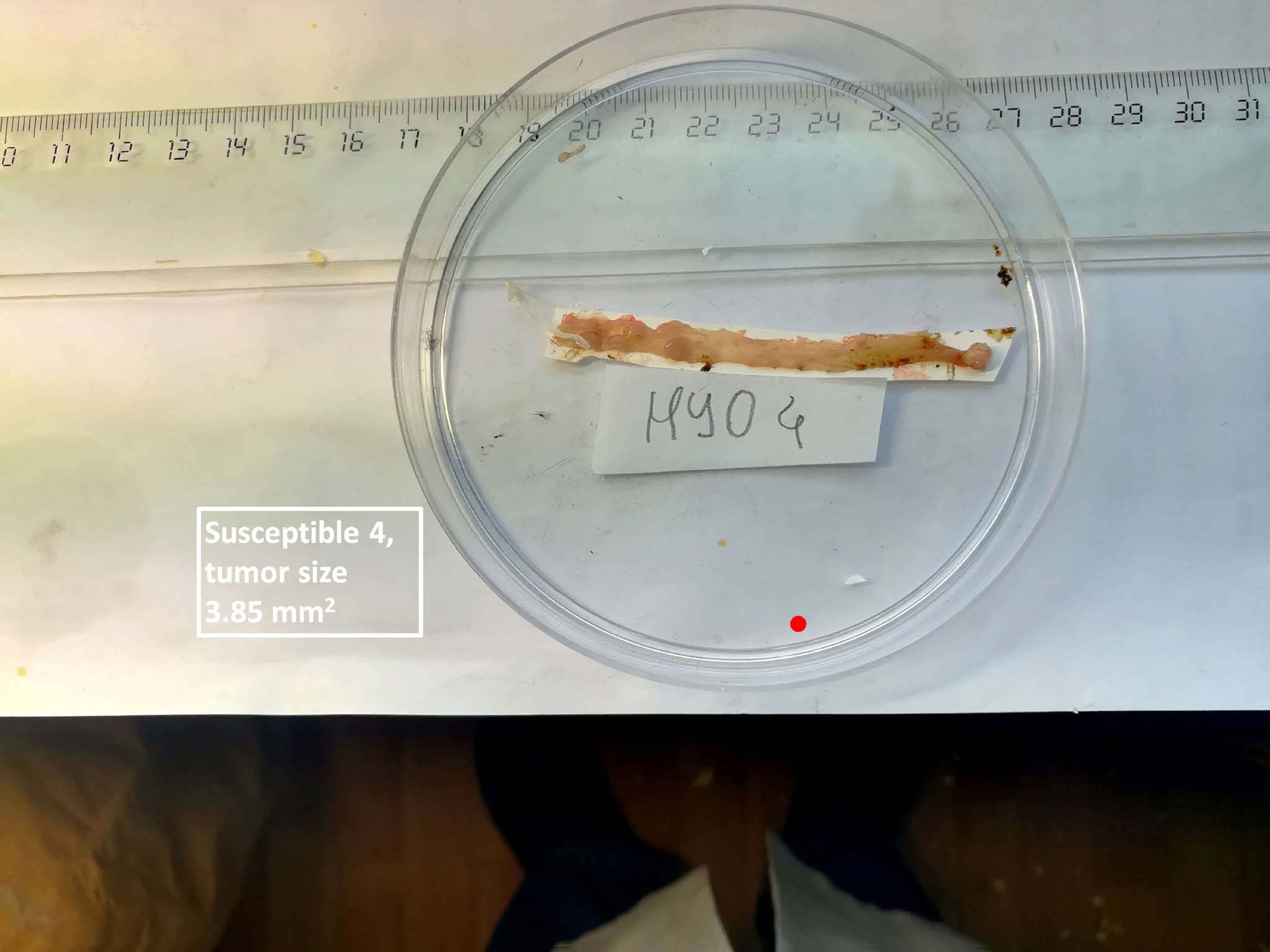
Susceptible 4, tumor size 3.85 mm^2

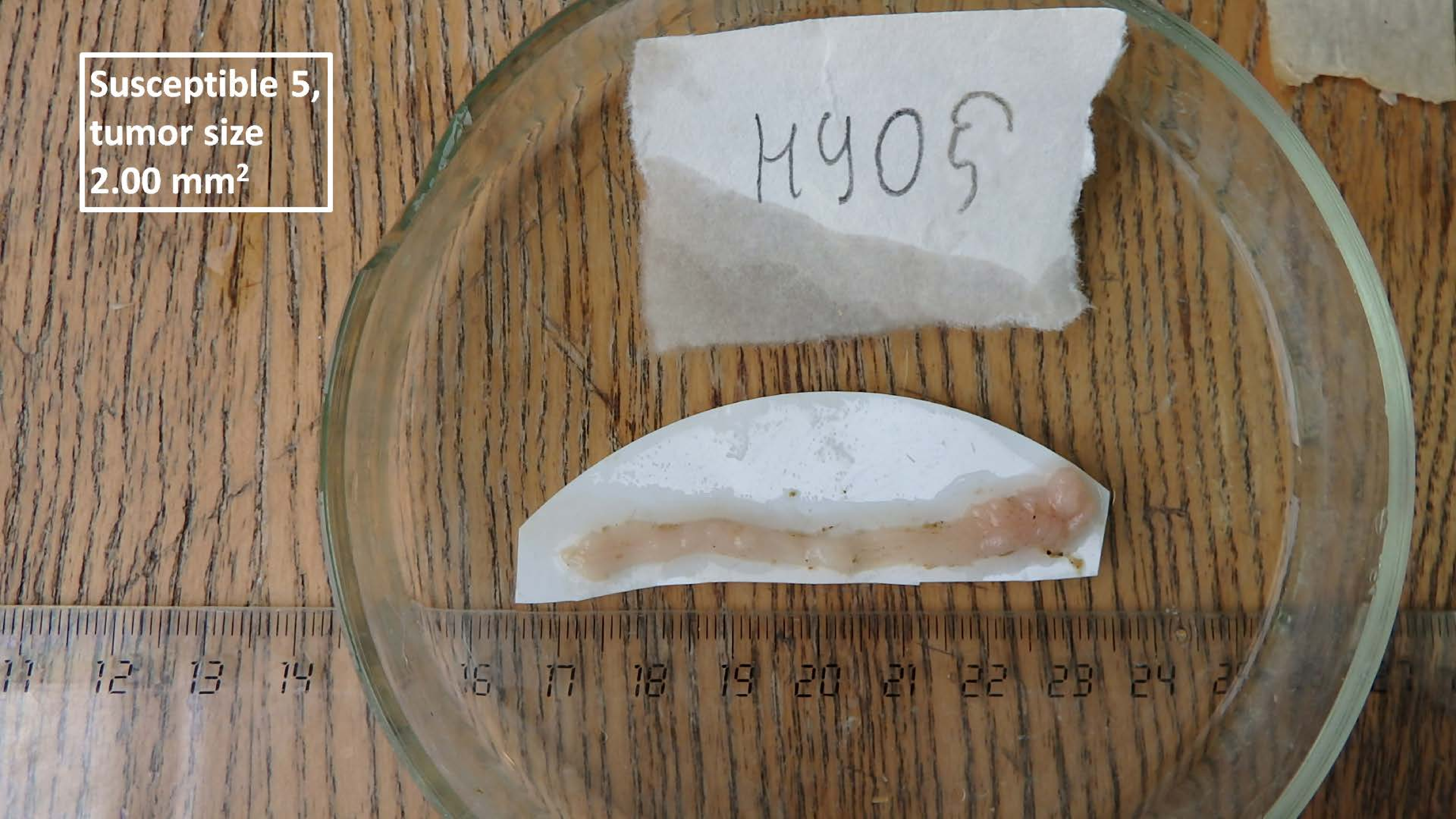
Susceptible 5,
tumor size
2.00 mm²
M405

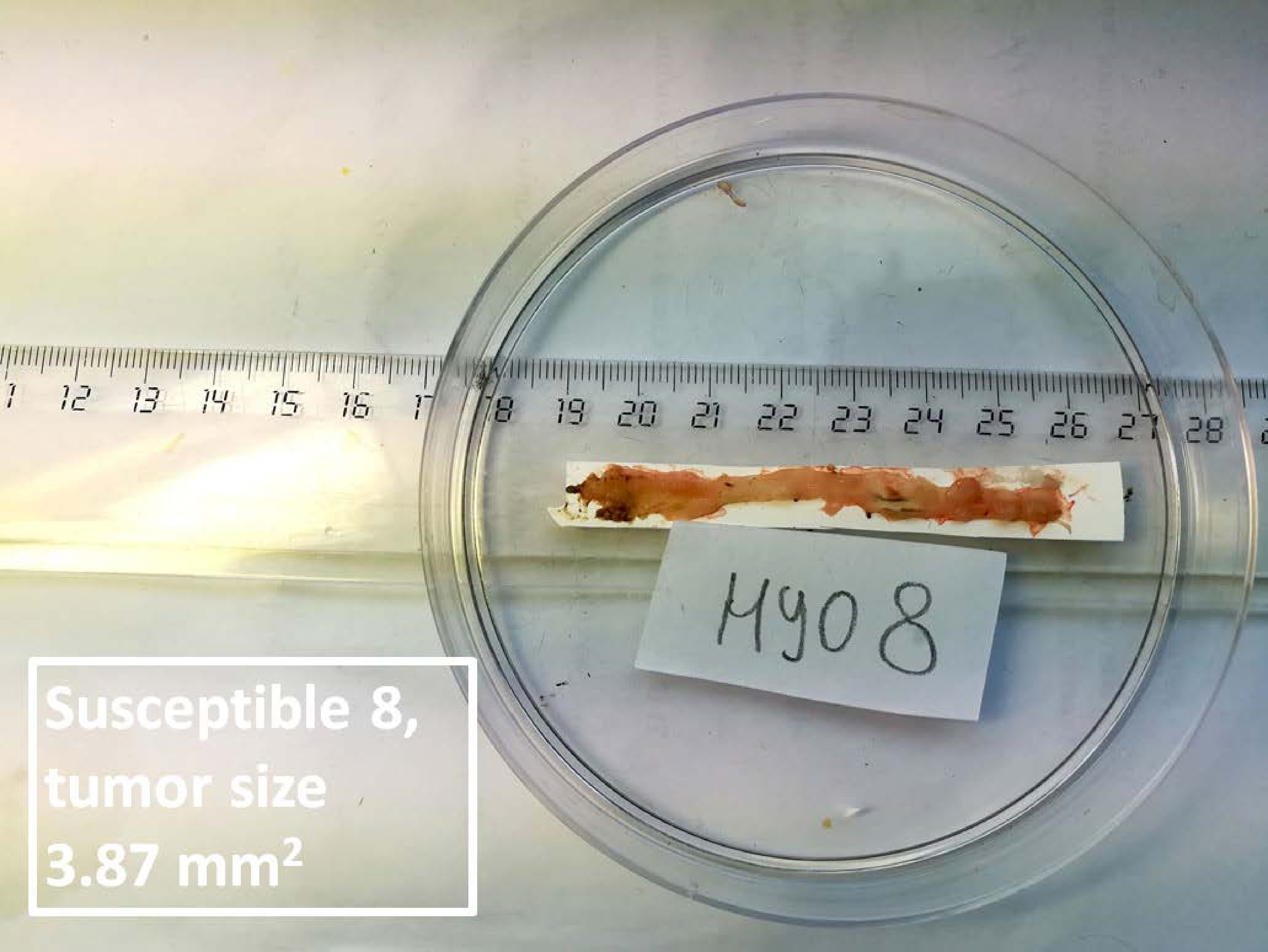
Susceptible 8,
tumor size
3.87 mm²
МУО 8

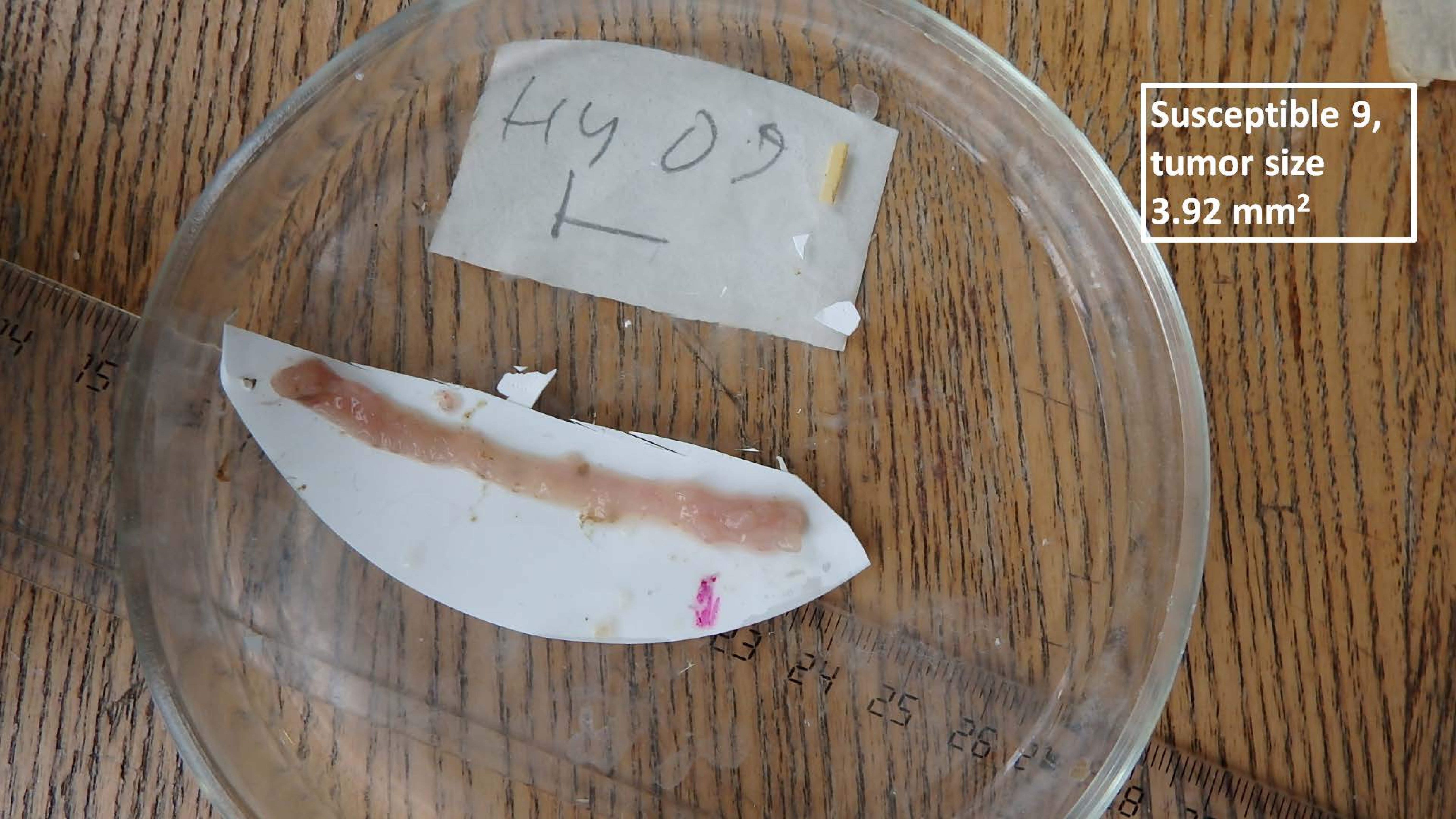
Susceptible 9,
tumor size
3.92 mm²

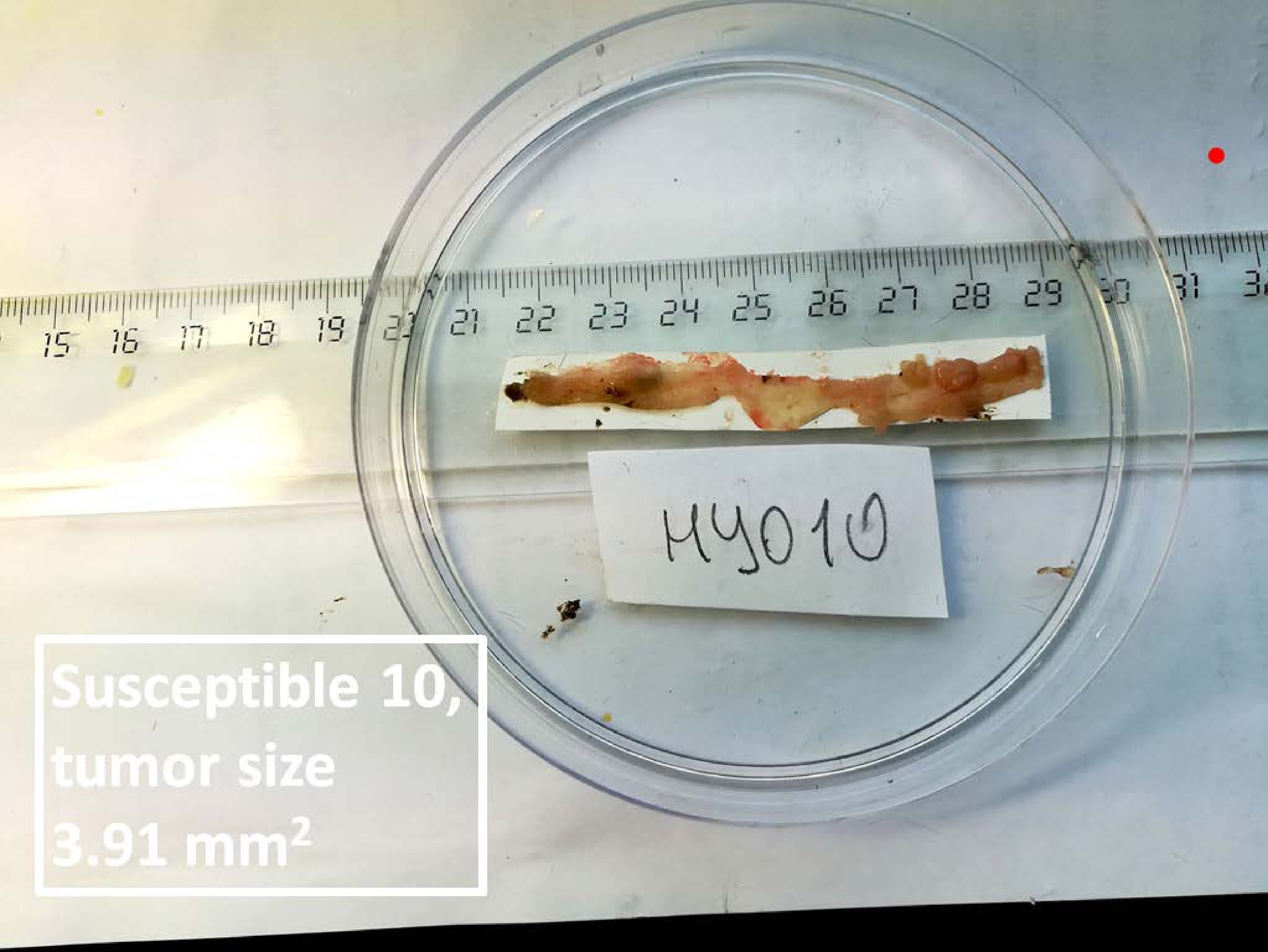
MY010
Susceptible 10, tumor size 3.91 mm^2

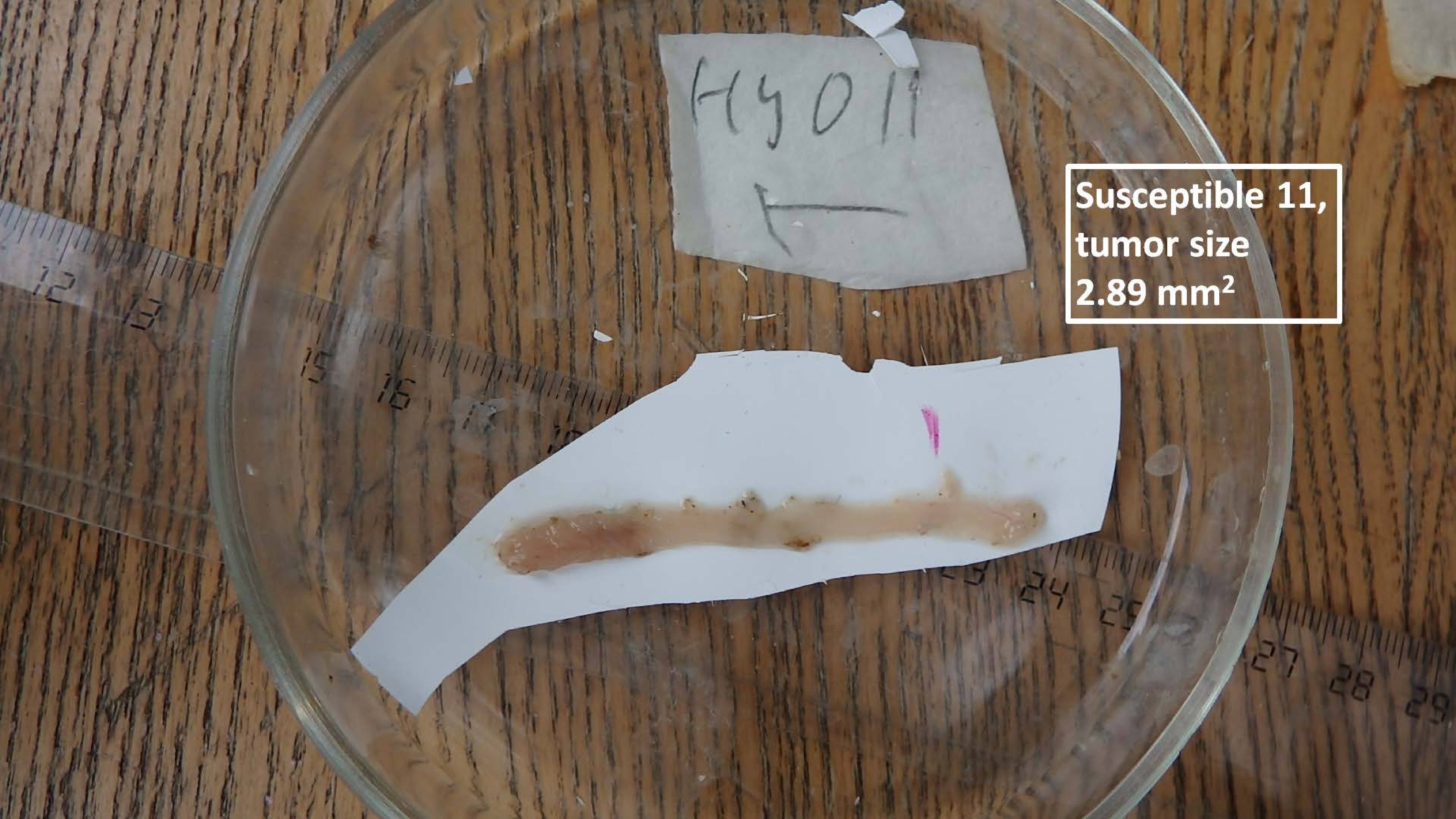
Susceptible 11, tumor size 2.89 mm²

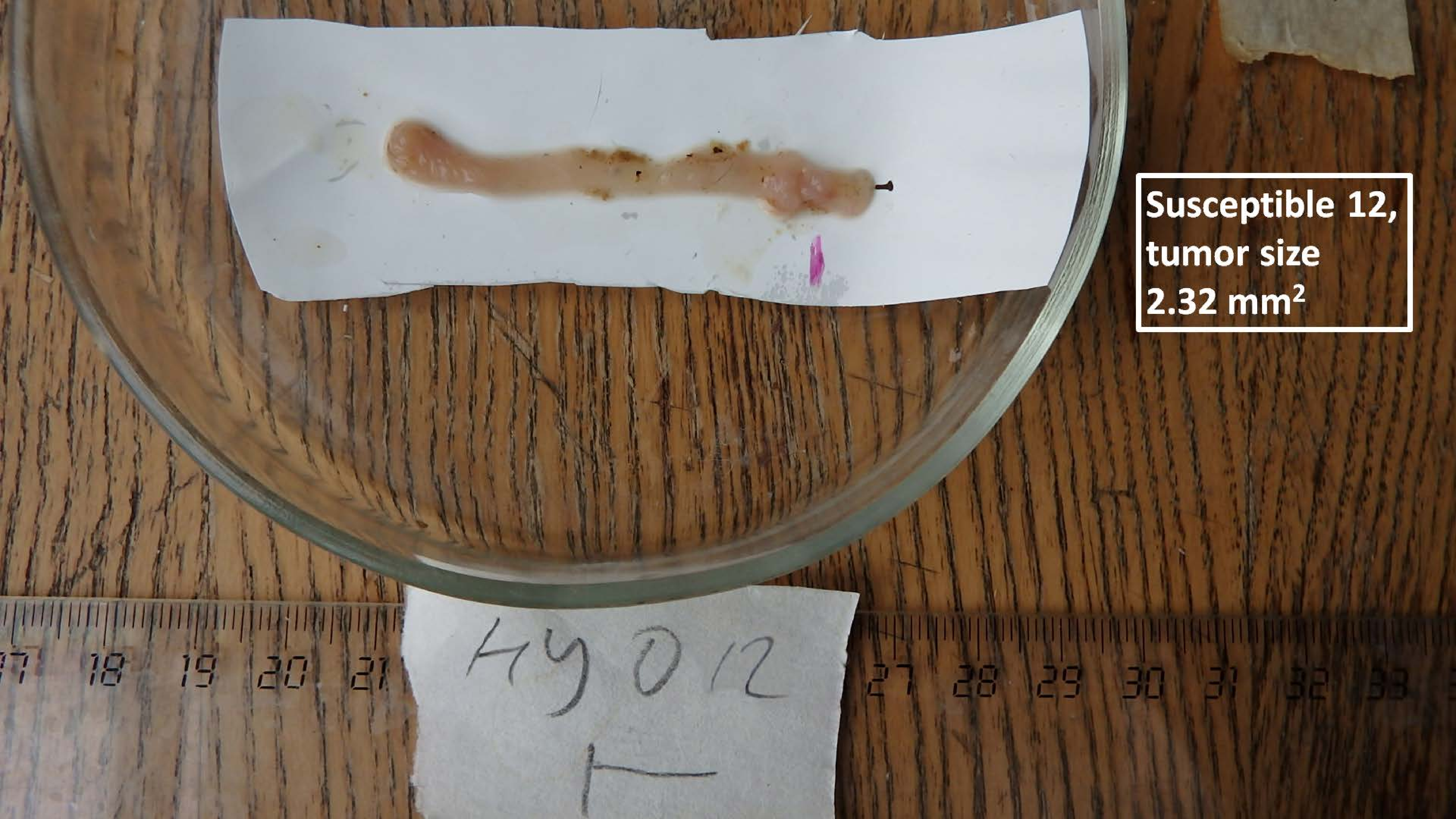
Susceptible 12,
tumor size
2.32 mm²
18 19 20 21
27 28 29 30 31 32 33

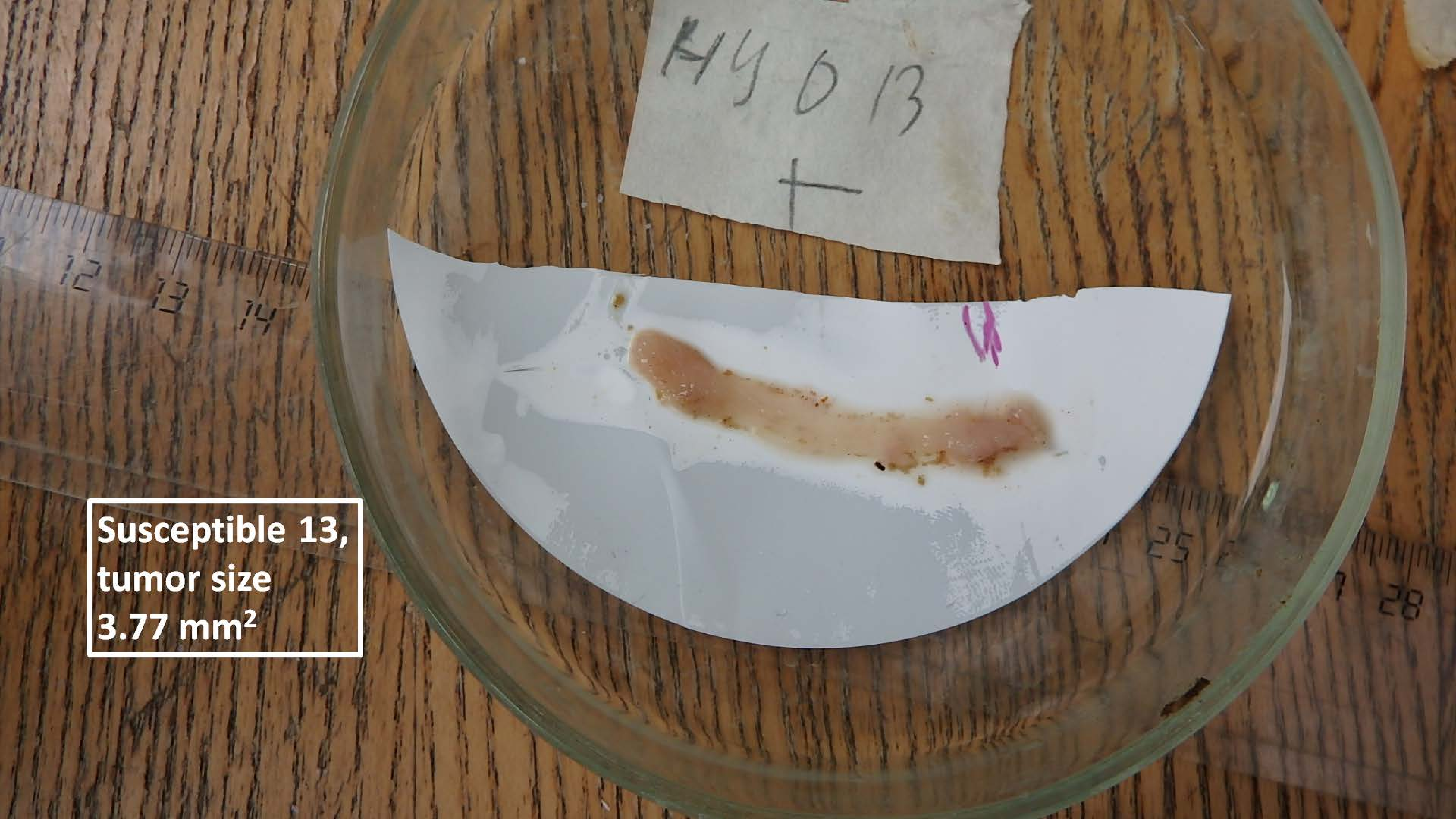
Susceptible 13,
tumor size
3.77 mm²

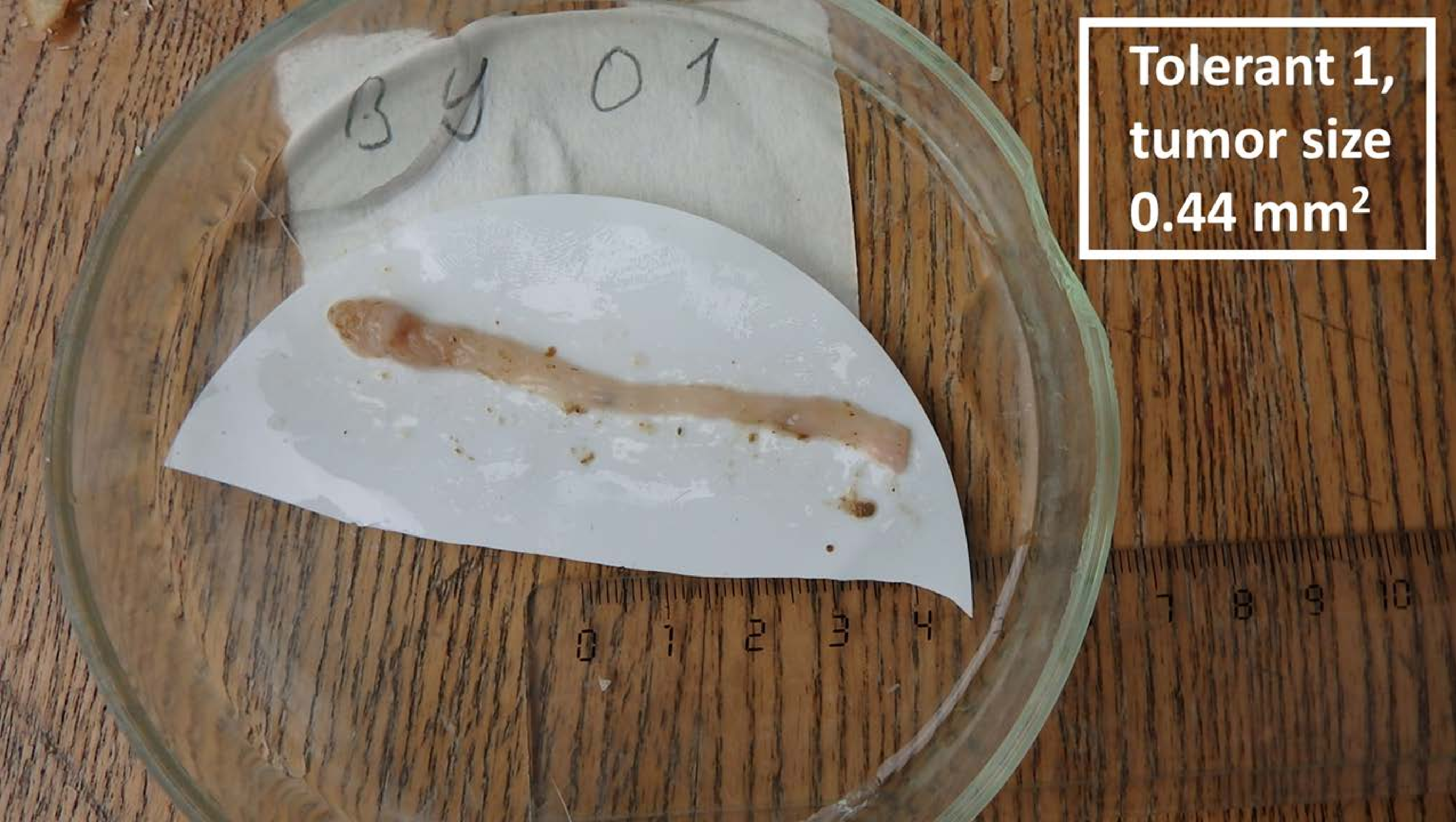
BY 01
Tolerant 1,
tumor size
$0.44\ mm^2$

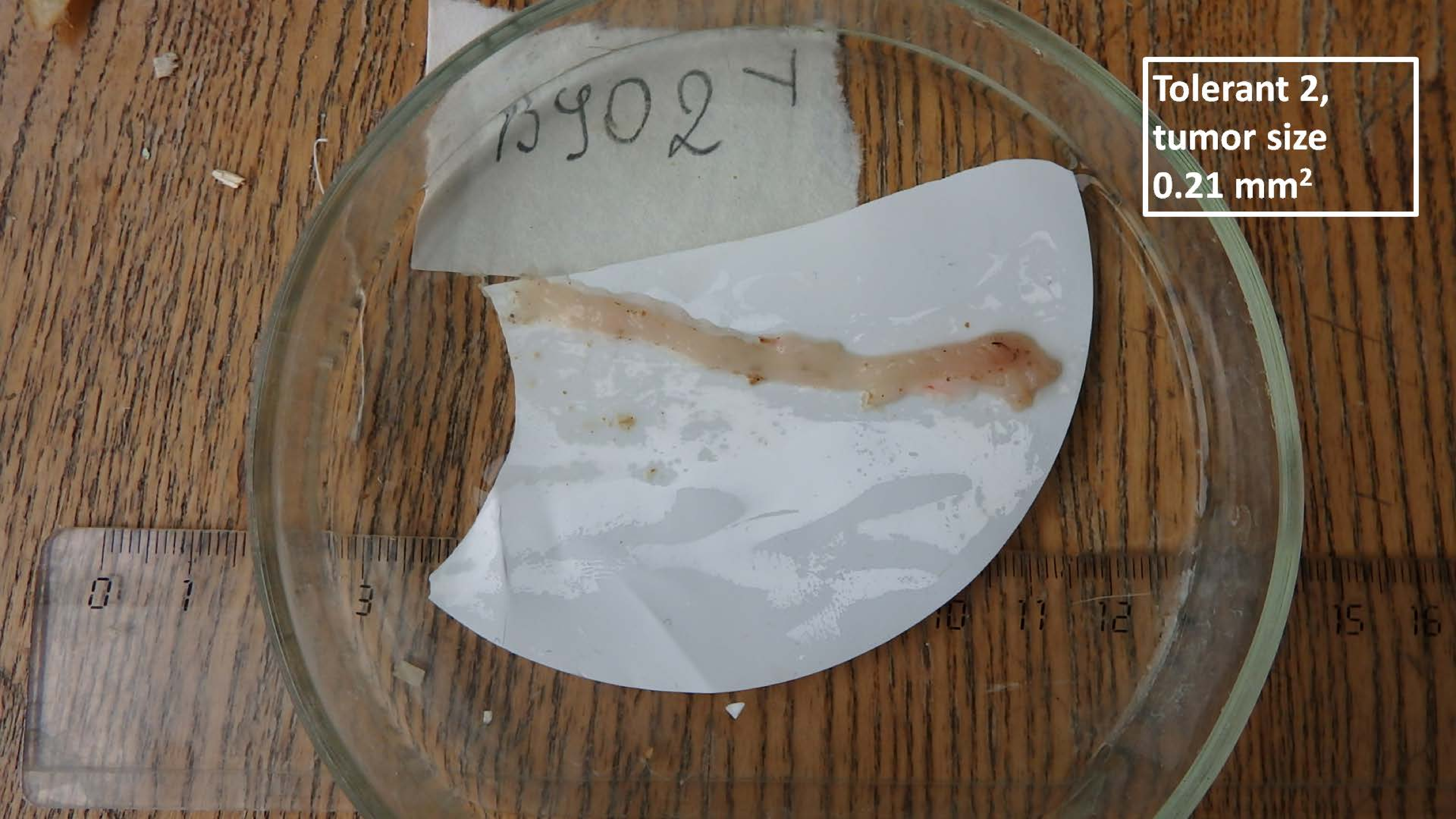
Tolerant 2,
tumor size
0.21 mm²

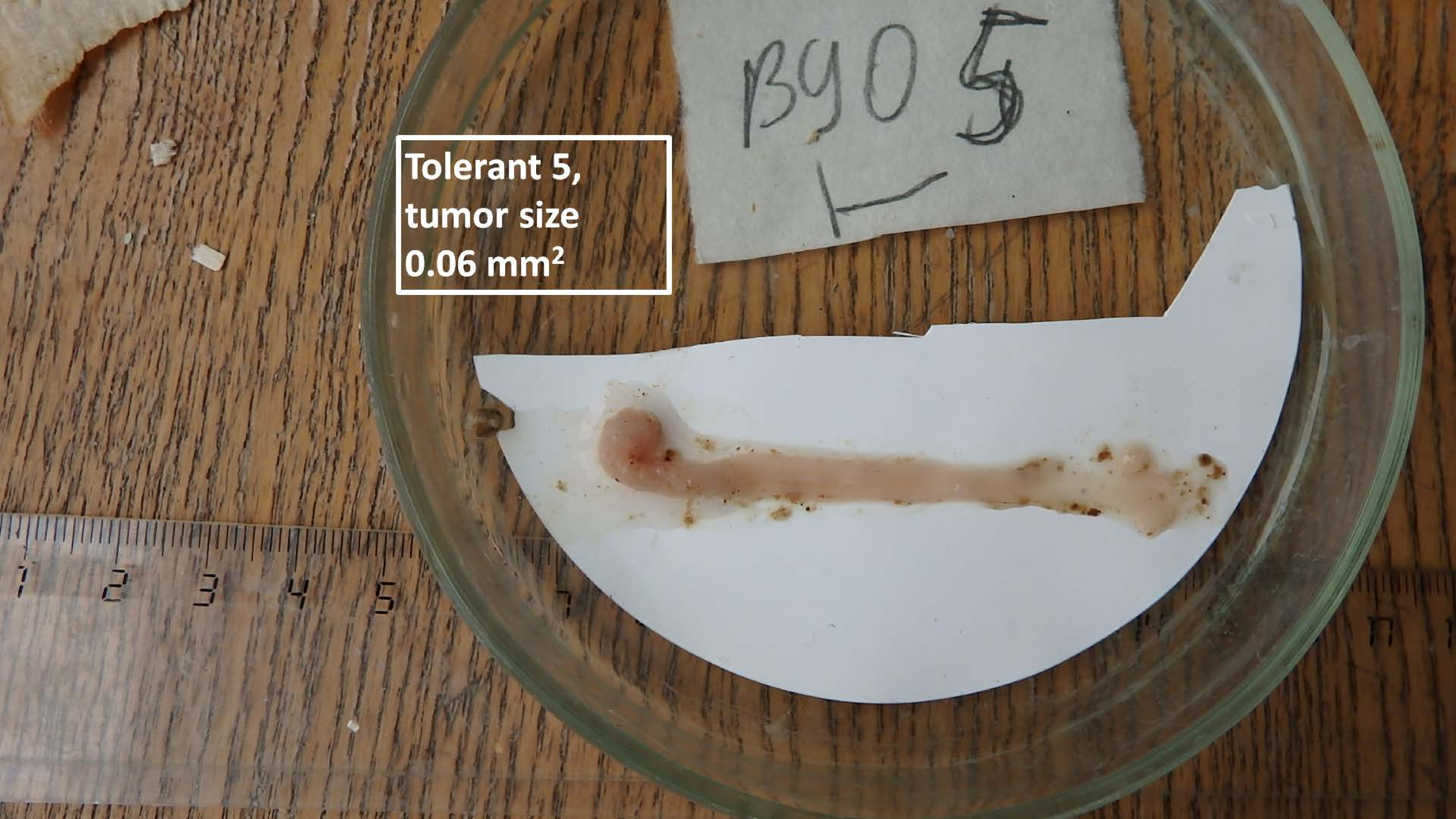
Tolerant 5,
tumor size
0.06 mm²
B905

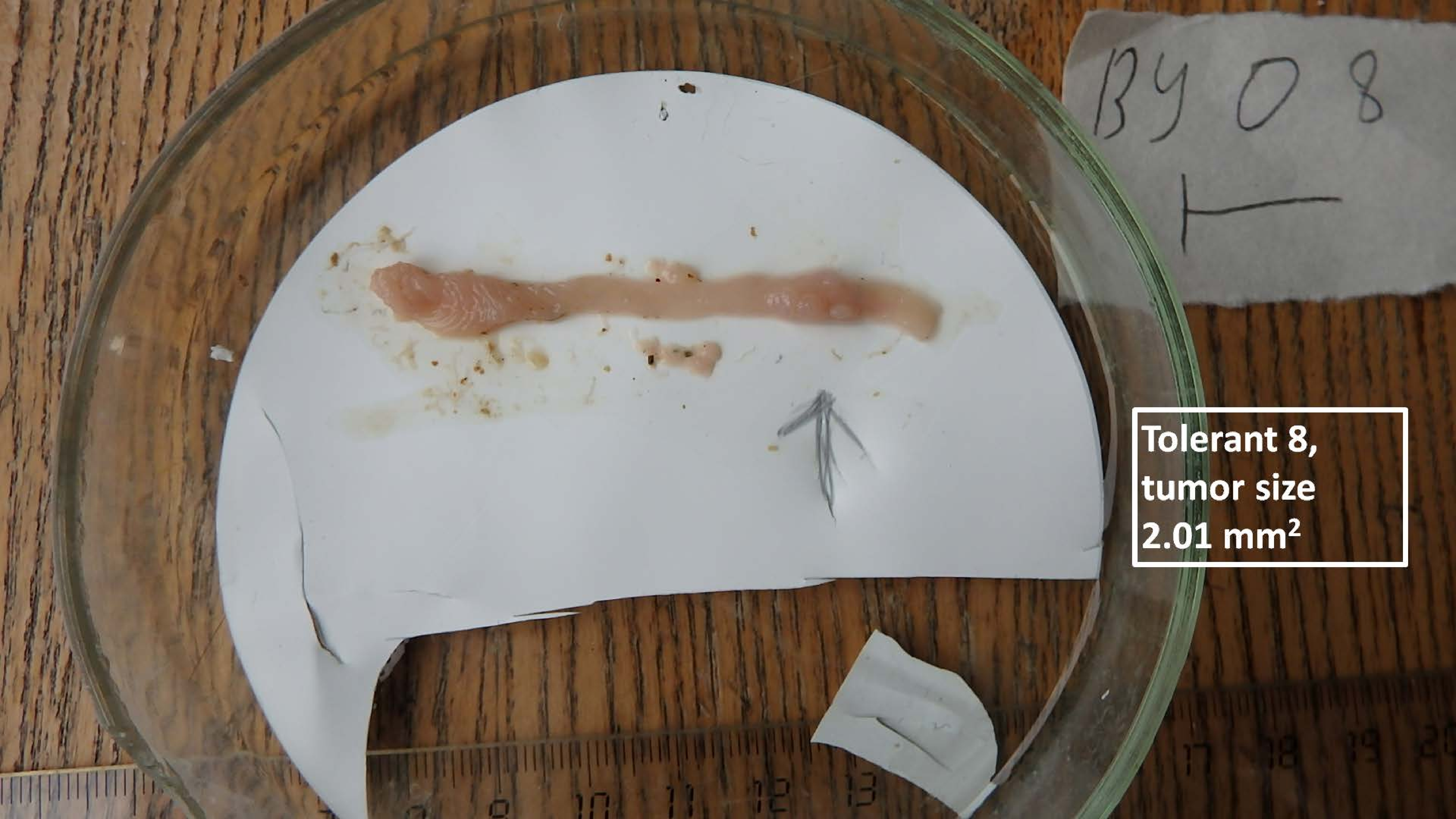
BY08
Tolerant 8, tumor size 2.01 mm²

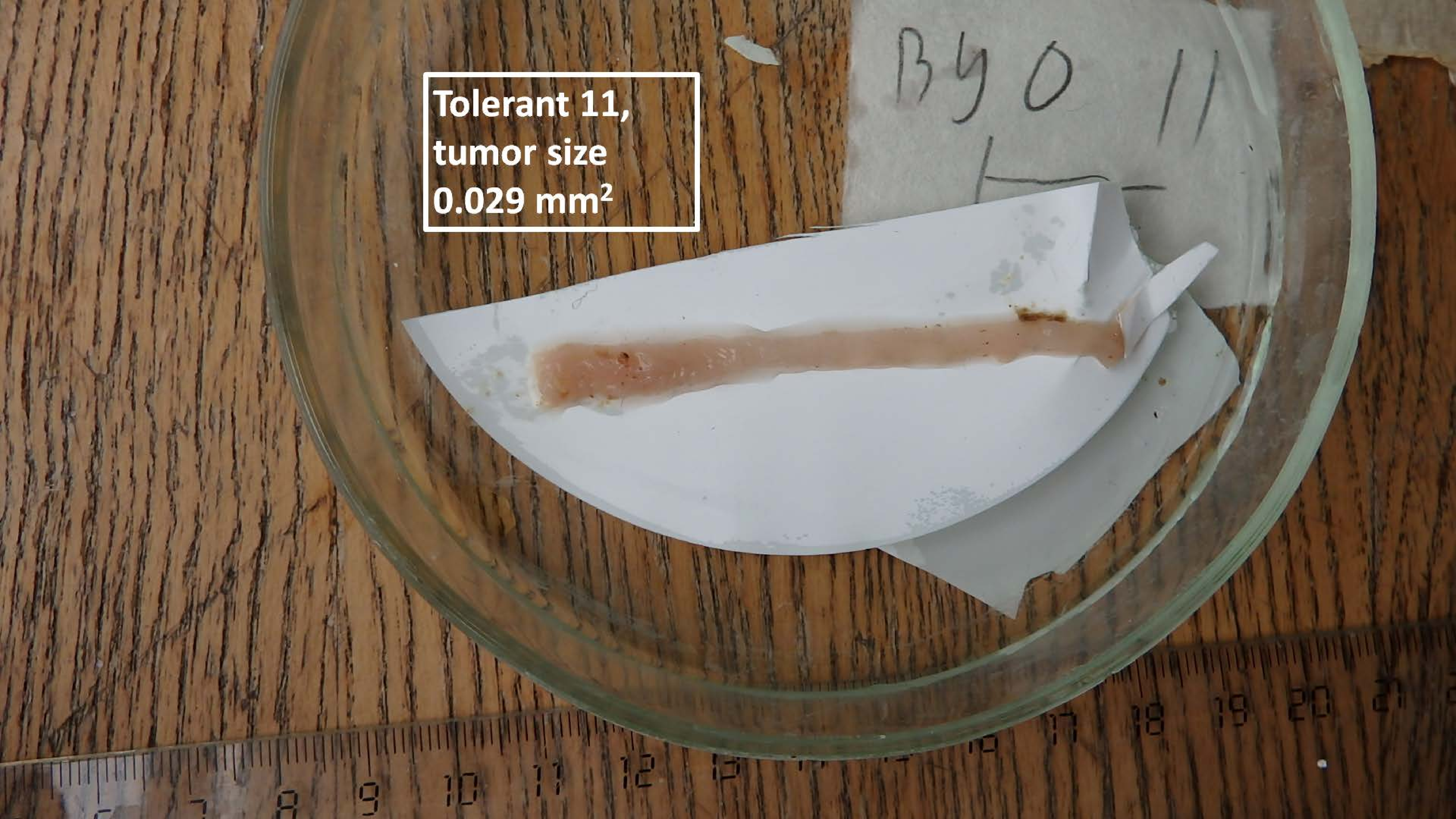
Tolerant 11,
tumor size
0.029 mm²
